# An exceptionally well-preserved herbaceous eudicot from the Early Cretaceous (late Aptian–early Albian) of Northwest China

**DOI:** 10.1093/nsr/nwab084

**Published:** 2021-05-04

**Authors:** Baoxia Du, Mingzhen Zhang, Bainian Sun, Aijing Li, Jing Zhang, Defei Yan, Sanping Xie, Jingyu Wu

**Affiliations:** Key Laboratory of Mineral Resources in Western China (Gansu Province), Lanzhou University, Lanzhou 730000, China; Northwest Institute of Eco-Environment and Resources, Chinese Academy of Sciences, Lanzhou 730000, China; Key Laboratory of Petroleum and Resources, Gansu Province, Lanzhou 730000, China; Key Laboratory of Mineral Resources in Western China (Gansu Province), Lanzhou University, Lanzhou 730000, China; Key Laboratory of Mineral Resources in Western China (Gansu Province), Lanzhou University, Lanzhou 730000, China; Key Laboratory of Mineral Resources in Western China (Gansu Province), Lanzhou University, Lanzhou 730000, China; Key Laboratory of Mineral Resources in Western China (Gansu Province), Lanzhou University, Lanzhou 730000, China; Key Laboratory of Mineral Resources in Western China (Gansu Province), Lanzhou University, Lanzhou 730000, China; Key Laboratory of Mineral Resources in Western China (Gansu Province), Lanzhou University, Lanzhou 730000, China

**Keywords:** eudicot, Early Cretaceous, Northwest China, Jehol Biota, *Gansufructus*, paleoecology

## Abstract

A fossil eudicot, *Gansufructus saligna* gen. et sp. nov., is reported from the Early Cretaceous (late Aptian–early Albian) of the Gansu Province, Northwest China, based on numerous well-preserved axes with attached leaves and infructescences. The leaves are alternate, short petiolate and linear-lanceolate with low rank pinnate to reticulate venation. The infructescences are loose panicles bearing fruits in different stages of maturity, each containing four partly free carpels borne in a whorled arrangement. Each carpel has three to five seeds borne along its ventral margin. The nature of the leaves and axes indicates a terrestrial, herbaceous habit. In general organization, *Gansufructus* is closely similar to the fruit-bearing axes of *Sinocarpus decussatus* from the Early Cretaceous Jehol Biota, as well as other more or less contemporaneous angiosperms from the Far East, which together provide evidence of diverse eudicot angiosperms of low stature colonizing areas close to environments of deposition.

## Teaser

An exceptionally well-preserved herbaceous eudicot was reported from the Early Cretaceous of Northwest China, providing important insights into the morphology, diversification and ecological environments of early angiosperms.

## INTRODUCTION

Angiosperms (flowering plants) represent the largest and most successful clade of vascular plants, with >350 000 extant species distributed all over the world [1,2], but their origin, evolution, early diversification, as well as the habitat preferences and ecology of early forms, are still poorly understood [3–5]. Some molecular studies suggest a pre-Cretaceous origin for angiosperms, perhaps Late Triassic [5–7], but there are no reliable fossil angiosperms in Triassic or Jurassic deposits [8,9], and the rise of angiosperms during the Early and mid-Cretacea has been regarded as a trigger for the Cretaceous Terrestrial Revolution (KTR) [10,11]. Rapid diversification of angiosperms in habit, morphology, anatomy, physiology and reproductive biology, may have been important in promoting the diversification of insects, amphibians, mammals, ferns and many other terrestrial organisms [12–15].

**Figure 1.**
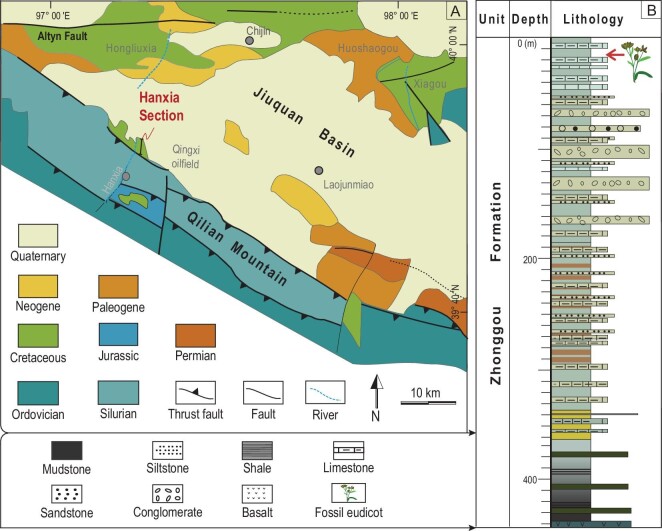
(A) Fossil locality of *Gansufructus saligna* gen et sp. nov. and (B) stratigraphic column of the Lower Cretaceous Zhonggou Formation in the Hanxia Section, showing the fossil bearing strata [31].

The Early Cretaceous terrestrial Jehol Biota is widely distributed in East Asia (northern China, southeastern Mongolia, Siberia, Korea and Japan), and is characterized by the *Eosestheria*-*Ephemeropsis*-*Lycoptera* assemblage [16]. Well-preserved and informative Jehol Biota fossils are particularly abundant in western Liaoning, eastern Heilongjiang, northern Hebei and southeastern Inner Mongolia, and include crucial specimens of feathered dinosaurs, early birds, eutherian mammals and early flowering plants [17–19]. Lower Cretaceous strata are also widely distributed in Northwest China, especially in the western part of Gansu Province, where numerous fossils document a rich Jehol fauna and flora [20] that includes fishes [21], turtles [22], insects [23], birds [24], dinosaurs [25] and plants [26–28], although no angiosperms have been described. Here, we report an early angiosperm from the late Early Cretaceous (late Aptian–early Albian, 115–112 Ma) [20,23,29–31] Zhonggou Formation of the Jiuquan Basin in Northwest China (Fig. 1). The fossil specimens are assigned to the eudicots clade based on the morphology of both vegetative and reproductive organs.

## SYSTEMATIC PALEONTOLOGY

Angiospermae

Eudicots

Incertae sedis

*Gansufructus* B. Du gen. nov.

**Etymology:** *Gansu,* after the region where the specimens were found, and *fructus*, Latin for fruit.

**Generic diagnosis**: Plant herbaceous, erect. Main axis slender, straight or slightly curved with alternating secondary axes. Leaves simple, elongated oval, lanceolate or ovoid-lanceolate shaped and alternately arranged on the branches. Leaf margin entire. Leaf apex acute. Leaf base decurrent and estipulate with short petiole. Leaf venation poorly organized, with pinnate lateral veins and reticulate tertiary veins. Infructescence open and paniculate. Ultimate branches bearing one to three terminal fruits. Gynoecium superior, basally syncarpous with four carpels fused or appressed proximally along their ventral surface for about half of their length. Carpels whorled, each in the axil of a small persistent tepal. Each carpel enclosing three to five anatropous ovules/seeds borne on linear placentae along the ventral suture. Seed oval or reniform.

*Gansufructus* is closely similar to *Sinocarpus* Leng et Friis, especially in the organization of the fruits that have four carpels united basally and arranged in a whorl. The two taxa are distinguished mainly by arrangement of leaves and twigs, size of the carpels and number of seeds. Carpels of *Gansufructus* are generally shorter, and each contains three to five seeds*,* whereas carpels of *Sinocarpus* typically contain ∼10–20 seeds. Branching of *Gansufructus* is alternate, whereas it is decussate in *Sinocarpus*. The general organization of the infructescences in *Hyrcantha* Krassilov et Vachrameev is very similar to *Gansufructus*, but *Hyrcantha* is distinguished from both *Gansufructus* and *Sinocarpus* by the apocarpous gynoecium.

**Plant Fossil Names Registry Number:** PFN001823 (for new genus)

*Gansufructus saligna* B. Du gen. et sp. nov.

Figures 2–4

**Figure 2.**
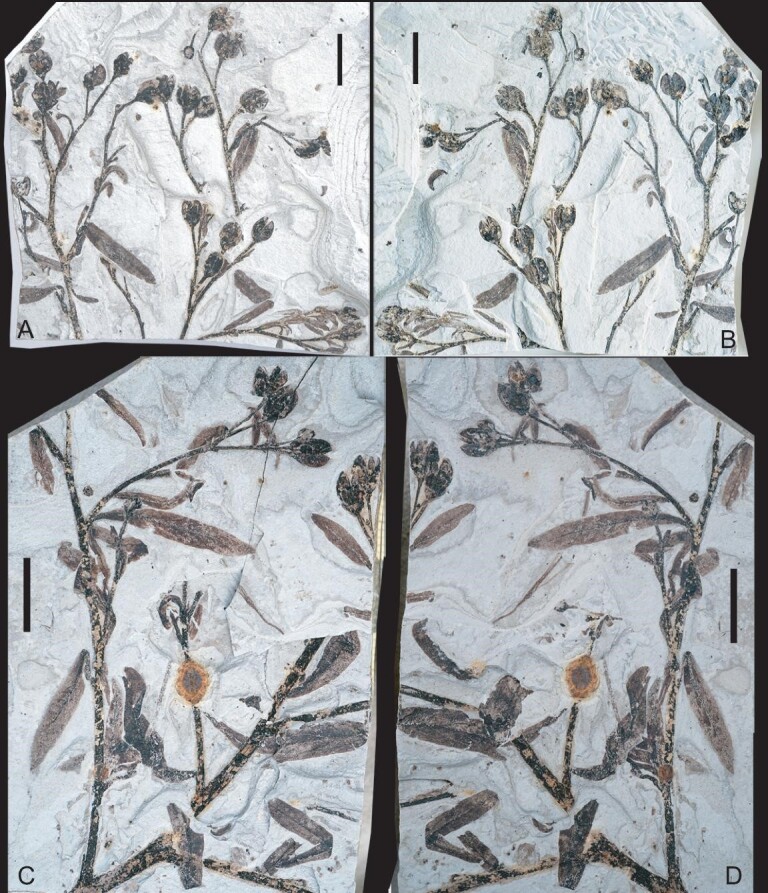
Infructescence axes bearing leaves of *G. saligna* gen. et sp. nov. (A and B) Part and counterpart of holotype of *G. saligna* gen. et sp. nov. showing leafy axes with infructescences and lanceolate leaves. Specimen JQ-2018-01(A and B). (C and D) Part and counterpart showing axes with alternate arranged branches and lanceolate leaves, as well as terminal fruits in different stages of maturity. Specimen JQ-2018-02(A and B). Scale bars: 1.0 cm.

**Holotype**: JQ-2018-01(A, B) (Fig. 2A and B)

**Figure 3.**
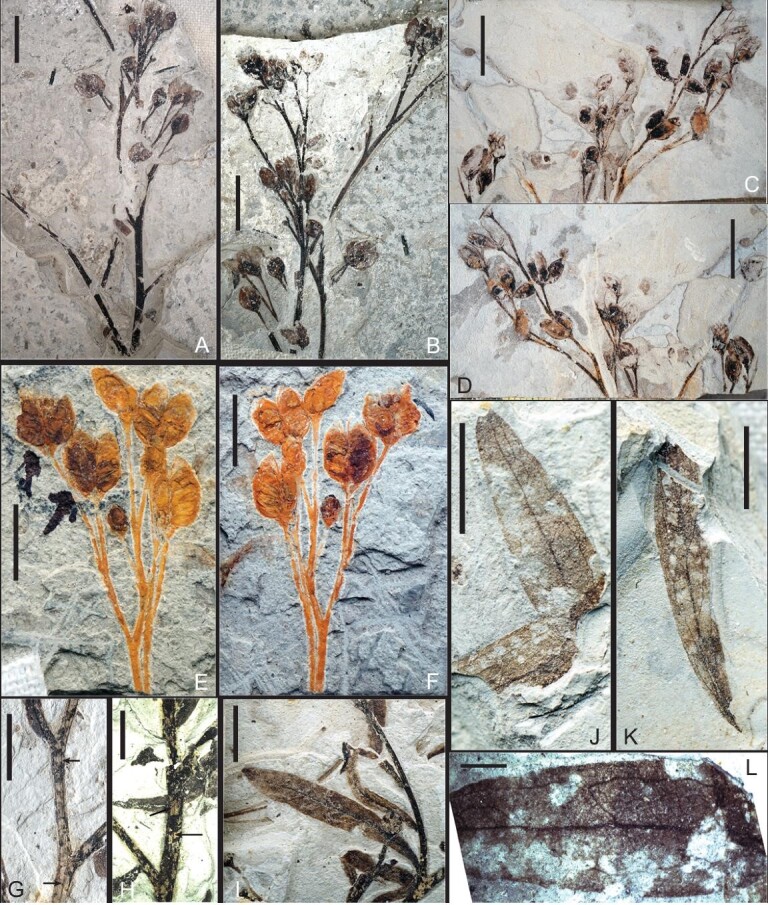
Infructescence, leafy shoots and isolated leaves of *G. saligna* gen. et sp. nov. (A and B) Part and counterpart showing paniculate and determinate inflorescences. Specimen JQ-2017-01(A and B). (C and D) Part and counterpart of an infructescence showing attached dehiscent fruits. Specimen JQ-2018-03(A and B). (E and F) Part and counterpart of a single determinate infructescence showing fruits with basally syncarpous carpels. Specimen JQ-2019-01(A and B). (G and H) Main axis showing alternate twigs, and the arrows showing the grooves or ribs on the stem surface. (I) Leaf showing the lanceolate shape and insertion on the stem. (J and K) Isolated leaves showing low rank venation. (J) Specimen JQ-2020-01. (K) Specimen JQ-2020-02. (L) Details of leaf venation showing the midrib, and poorly organized pinnate lateral vein and irregularly reticulate tertiary venation. Scale bars: (A–D) 1.0 cm; (E–K) 0.5 cm; (L) 0.1 cm.

**Paratypes**: JQ-2017-01(A, B), JQ-2018-02(A, B), JQ-2018-03(A, B), JQ-2019-01(A, B), JQ-2020-01 and JQ-2020-02 (Fig. 2C and D and Fig. 3)

**Etymology**: *saligna*, from the willow-shaped leaves (*saligna*, Latin for ‘willow’)

**Locality and horizon:** Laojumiao of Jiuquan City, western Gansu Province, Northwest China, uppermost part of the Zhonggou Formation, Hanxia Section; Early Cretaceous (late Aptian–early Albian)

**Plant Fossil Names Registry Number:** PFN001822 (for new species)

**Specific diagnosis**: As for the genus

**Description:** The plant was fossilized at fruiting stage and preserves the terminal part of a simple or more complex infructescence with attached leaves (Figs 2 and 3A–F). Plant erect, herbaceous, three to four times branched. Branches alternate and are predominantly at angles of 30–45° (Figs 2 and 3A–H). The main axis is slender and lightly striated, ∼2–4 mm wide, with longitudinal grooves or ribs on the surface (Fig. 3G and H); secondary branches are ∼1–1.2 mm wide, and tertiary branches are ∼0.5 mm wide (Figs 2, 3A and B). Ultimate branches bear one to three terminal fruits (Figs 2 and 3A–F). Leaves are simple, symmetrical, deciduous or persistent and alternately arranged. They vary in size, typically being ∼1–2.5 cm long and 0.2–0.4 cm wide, but are larger toward the base of the plant (Fig. 2). Leaves are narrow-ovate, lanceolate or ovoid-lanceolate in shape (Figs 2 and 3I). Leaf apex is acute, and the leaf margin is entire (Figs 2 and 3I–K). Leaf base is decurrent with a short petiole (Fig. 3I).

Leaf venation is poorly organized, pinnate to reticulate (Fig. 3I). The primary vein is prominent and straight, or slightly curved, and extends from the leaf base to the apex (Fig. 3I–K). Secondary veins are pinnate, arcuate and arise from the mid-vein alternately, each at an angle of 30–45°, but do not reach the leaf margin (Fig. 3J–L). Inter-secondary veins are shorter than secondary veins, extend from the mid-vein, and often fuse with the vein loops formed by the secondary veins. Tertiary veins are reticulate and oblique to the main course of the secondary veins (Fig. 3L).

**Figure 4.**
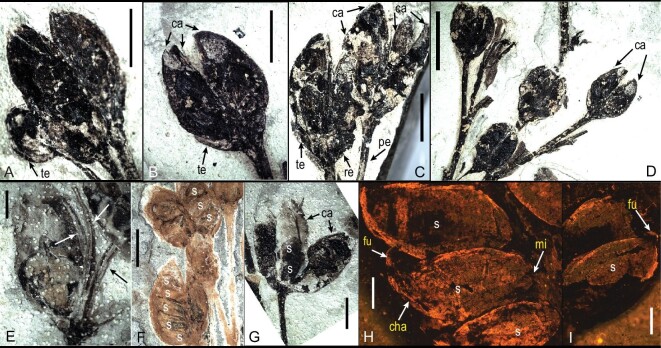
Fruit, carpel and seed morphology of *G. saligna* gen. et sp. nov. with stereo and fluorescence microscopy. (A) Three fossil fruits at different stages of maturity, one large and two others that are smaller. The black arrow shows the remains of tepal (te) at the base. (B) Single fruit with dehiscent carpels (ca), showing the persistent tepal (te) below the carpels. (C) Dehiscent fruits with four carpels (ca) borne in a whorl, showing the convex receptacle (re), persistent tepal (te) and slender pedicel (pe). (D) Fruits showing carpels (ca) closely adherent basally for about half or more of their length. (E) Fruit showing dehiscence of carpels. The arrows show the dehiscent carpels along the ventral side. (F) Fruits showing basally syncarpous carpels containing three to five seeds (s) attached along the ventral suture. (G) Dehisced fruit showing elongated elliptic carpels arranged in a whorl, containing asymmetric, oval to elongated ovoid seeds (s). (H and I) Anatropous seeds (s) inside the carpel along the ventral suture under fluorescence microscopy, showing the funiculus (fu), chalaza (cha) and micropyle (mi). Scale bars: (A–C), (F and G) 2 mm; (D) 5 mm; (E) 1 mm; (H and I) 400 μm.

The infructescence is open, paniculate and determinate, bearing terminal fruits at the apex of an elongated pedicel (Figs 2, 3A–F, 4A–D and G). Pedicels are slender, ∼3.5–5.5 mm long, and 0.5–0.6 mm wide (Figs 3A–F, 4C, D and G). The gynoecium is basally syncarpous, and the ovary is superior (Figs 2, 3A–F and 4A–G). The fruits consist of four carpels arranged in a whorl on a convex receptacle (Fig. 4A–D). Receptacles are distinct and slightly expanded, up to ∼2 mm long and 1.8 mm wide (Fig. 4C and D). A small and persistent tepal subtends each carpel (Fig. 4A–C). There are no other remains of either perianth or stamens, and it is unknown whether the flowers were unisexual or bisexual.

Prior to dehiscence, the closed fruits are elliptic or subglobose in shape, ∼2–3 mm long and 2–3 mm wide (Fig. 4A). After dehiscence along the ventral suture, the fruits are ∼4–5.5 mm long and 3–4.5 mm wide (Fig. 4A–G). In most fruits, the carpels are fused basally for about half or more of their length (Fig. 4A–D). Some are completely dehisced, and the elongated elliptic-shaped carpels are arranged in a whorl (Fig. 4E and G). The carpels are asymmetric with mucronate apices, ∼4.5–5.5 mm long and 2–2.5 mm wide (Fig. 4B–D, F and G), and dehisce along the ventral side (Fig. 4E). Each carpel contains ∼3–5 ovules/seeds arranged longitudinally on linear placentae along the ventral suture of the carpel, both in the free and fused portion of the fruit (Fig. 4F).

Seeds are tightly packed in the carpels with their margins sometimes overlapping (Fig. 4F–I). Seeds vary in size and morphology. They are ∼1.5–1.8 mm long and 0.8–1.1 mm wide, asymmetrical and oval to elongated ovoid or reniform in shape (Fig. 4F–I), slightly pointed in the hilar region and rounded to truncate in the chalaza region (Fig. 4F–I). Seeds are anatropous, with chalaza opposite to the funiculus, and the micropyle situated at the base of the funiculus (Fig. 4H and I).

**Figure 5.**
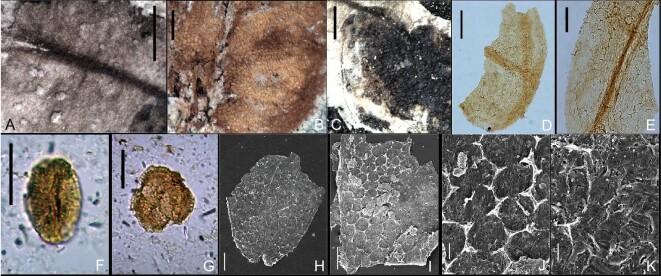
Cuticular structure of *G. saligna* gen. et sp. nov. and eudicot pollen grains from the fossil bearing strata. (A) Surface of carpel under stereo microscopy, showing the polygonal or elongated rectangular-shaped cells. (B and C) Cuticular structures of seeds under stereo microscopy, showing the pentagonal- and hexagonal-shaped cells. (D and E) Cuticles of seeds under light microscopy, showing two cuticular layers. (F and G) Eudicot pollen grains from the fossil bearing horizon of uppermost Zhonggou Formation, Hanxia Section. (H–K) Cuticular structures of seeds under scanning electron microscopy, showing pentagonal- and hexagonal-shaped cells and irregularly curved anticlinal walls, as well as transverse parallel ribs and grooves on the periclinal walls. Scale bars: (A–C) 0.5 mm; (D and E) 200 μm; (F and G) 20 μm; (H and I) 100 μm; (J) 20 μm; (K) 10 μm.

Epidermal cells on the carpels are irregular, polygonal or elongated rectangular, ∼50 μm long and 15–20 μm wide (Fig. 5A). Two epidermal layers are visible on the seeds. The inner layer is formed by pentagonal and hexagonal cells arranged in longitudinal rows that radiate from the chalaza towards the micropylar end of the seed (Fig. 5B and C). The cells become larger near the micropylar area, being ∼80–110 μm long and 50–80 μm wide, and narrowly elongated towards the chalaza part of the seeds (Fig. 5B–E, H and I). The cells of the outer layer are elongated (Fig. 5D and E), with irregularly curved anticlinal walls (Fig. 5D, E and H–J), and transverse ribs and grooves on the periclinal walls (Fig. 5I–K).

## DISCUSSION

**Figure 6.**
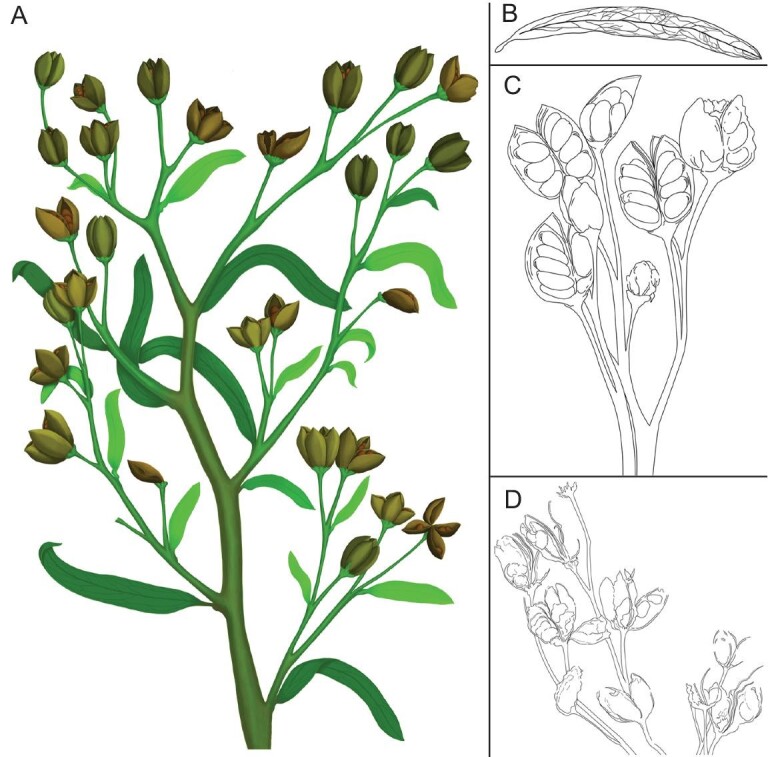
Reconstruction of *G. saligna* gen. et sp. nov. (A) Overview of *G. saligna* gen. et sp. nov. (B) Line diagram of a leaf showing lanceolate shape, short petiole, acute apex, entire margin and poorly organized venation. (C) Line diagram of fossil specimen JQ-2019-01(B), showing the basally syncarpous carpels enclosing three to five anatropous seeds. (D) Line diagram of fossil specimen JQ-2018-03(B), showing the dehiscent fruits with carpels arranged in a whorl (diagram by Mingchen Zhang).

*Gansufructus saligna* gen. et sp. nov. is reconstructed as a small, slender plant with flexible stems, delicate leaves and paniculate infructescences (Fig. 6A). The pinnate-reticulate low rank leaf venation (Fig. 6B) together with partly syncarpous gynoecium and several completely enclosed seeds (Fig. 6C and D) securely place this ancient plant within the angiosperms. In addition, general morphological features of *G. saligna*, including alternate phyllotaxis, pinnate-reticulate leaf venation, partly apocarpous gynoecium and fruit with four carpels arranged in a whorl, indicate an affinity among the eudicots.

Eudicots appeared early in the diversification of angiosperms, as evidenced by worldwide discoveries of tricolpate pollen grains as well as fossil flowers, fruits and leaves from late Barremian and early Albian strata [5,6,32–34]. However, few macro-fossils of eudicots have been reported from Albian or earlier rocks, and very few are known from both vegetative and reproductive organs. Among the several fossil records (Table 1), the infructescence of *Sagaria cilentana* is dichasium, and fruits are cup-shaped, composed of at least three follicles, and leaves are lobate [35]*. Achaenocarpites capitellatus* is characterized by stipulate, basically ternate, pinnatisect or three-lobed leaves and the reproductive structure is preserved as a head of achenes consisting of ∼16 radially spreading achenes. *Ternicarpites floribundus* possesses pinnatisect leaves and an apocarpous gynoecium of two to five, usually three, follicular carpels [36,37]. *Leefructus mirus* is characterized by three-lobed leaves, and its fruit have five pseudo-syncarpous elongated carpels [17]. The fossil specimens described in this paper are distinguished from all these fossils by their simple and lanceolate leaves, paniculate and determinate infructescence with dehiscent fruits composed of four basally syncarpous carpels, each enclosing three to five oval ovules/seeds.

**Table 1.** Morphological comparisons of early eudicot fossils known from both vegetative and reproductive organs.

Characters		*Sagaria cilentana*	*Achaenocarpites capitellatus*	*Ternicarpites floribundus*	*Leefructus mirus*	*Sinocarpus decussatus*	*Hyrcantha karatscheensis*	*Gansufructus saligna*
Infructescence type		Dichasium	Head of achenes	Unknown	Single fruit and axillary	Paniculate and determinate	Paniculate	Paniculate and determinate
Fruit	Size	11 mm long and 6 mm wide	3.6–3.9 mm in diameter	6–8 mm long	6 mm long and 4 mm wide	13–15 mm long and 6–8 mm wide	7 mm long and 3 mm wide	4–5.5 mm long and 3–4.5 mm wide
	Shape	Polycarpous, cup-shaped receptacle, basally fused, and dehiscent at the top	Globose	Polycarpous, ternate follicetum, and completely dehiscent	Polycarpous, loosely fused at its basal two-thirds, with a flattened receptacle	Polycarpous, basically united, and upper-middle part dehiscent	Polycarpous and completely dehiscent	Polycarpous, elliptic or subglobose, basically united, and upper-middle part dehiscent
Carpel or achene	Size	11 mm long and 3 mm wide	1.5–1.8 mm long	6–8 mm long and 2–3 mm wide	6 mm long and 0.5 mm wide	9–12.5 mm long and 1.5–3 mm wide	7 mm long and 3 mm wide	4.5–5.5 mm long and 2–2.5 mm wide
	Number	≥3	≥16	2 to 5, mostly 3	5	2 to 4	3 or 5	4
	Shape	Fused at its basal three-quarters, and distal tips mucronate	Obovate and minutely mucronate	Elongated	Elongated tips, and loosely fused at its basal two-thirds	Elongated elliptic	Ascidial	Elongated elliptic and asymmetric
	Arrangement	Basally syncarpous and whorled	Radially spreading	Apocarpous and whorled	Pseudo-syncarpous	Basally syncarpous and whorled	Apocarpous and whorled	Basally syncarpous and whorled
Seed	Size	Unknown	∼1 mm long	Unknown	Unknown	2 mm long and 1 mm wide	Unknown	1.5–1.8 mm long and 0.8–1.1 mm wide
	Number per carpel	Unknown	1	Unknown	Unknown	10–20, in two rows	Unknown	3–5
	Shape	Unknown	Ovate	Elliptical	Unknown	Ovate and asymmetric	Unknown	Oval to elongated ovoid or reniform, asymmetric
	Arrangement	Unknown	Anatropous	In rows along the abaxial suture	Unknown	Anatropous, along linear placentae	Unknown	Anatropous, along the ventral suture
Leaf	Shape	Lobed and petiolate	Basically ternate, pinnatisect or three-lobed	Pinnatisect of opposite decurrent lobes	Simple and deeply trilobate	Ovate or narrowly ovate, acute apex, rounded base and serrate leaf margin	Pinnately compound and ternate	Simple and lanceolate, acute apex, decurrent base, and entire margin
	Arrangement	Alternate or helical	Opposite	Unknown	Clustered	Unknown	Unknown	Alternate
Place		Southern Italy	Eastern Russia	Eastern Russia	Southern Italy	Northeastern China	Western Kazakhstan	Northwestern China
Age		Middle Albian	Early-Middle Albian	Early-Middle Albian	Middle Albian	Late Barremian	Middle Albian	Late Aptian-Early Albian
Reference		[35]	[36,37]	[36,37]	[17]	[38,39,44]	[40]	This paper

*Gansufructus* closely resembles *Sinocarpus* and *Hyrcantha* in gross morphology (Table 1), as well as in the paniculate infructescence and polycarpous fruits. They may be closely related despite showing different branching types and leaf characteristics. *Sinocarpus decussatus* differs from *G. saligna* by its decussate phyllotaxis, ovate or narrow-ovate leaves with serrated margin, and its greater number of seeds per carpel (10–20 ovules/seeds in *Sinocarpus* versus ∼3–5 in *Gansufructus*) [38,39]. *Hyrcantha karatscheensis* is distinguished by its apocarpous gynoecium, and the terminal fruiting units that consist of three or five carpels [40].

Among many extant families of eudicots, the combined characteristics of *Gansufructus*, such as simple, lanceolate and alternately arranged leaves with entire margins and pinnate-reticulate venation, as well as subglobose and polycarpous fruits, suggest a systematic position among the basal grade of eudicots or the basal core eudicots as also suggested for *Sinocarpus* [38,39]. In particular, there are similarities to extant Ranunculaceae, Myrothamnaceae and Buxaceae, but *Gansufructus* differs from all three of these families. Ranunculaceae is characterized by spiral phyllotaxis, simple to compound leaves and apocarpous gynoecium [41]. Myrothamnaceae is distinguished by decussate phyllotaxis, sessile leaves and catkin-like inflorescences [42]. Buxaceae is distinct in having fruits with two to three carpels, each of which always carries only two ovules [43].

Palynological preparations made from the fossil specimens failed to provide pollen associated with *Gansufructus*. However, poorly preserved tricolpate pollen grains, typical of eudicots, do occur in the fossil-bearing strata, and the pollen assemblage is mainly dominated by grains assignable to the extinct pollen genus *Retitricolpites* (Fig. 5F and G). Previous palynological analyses have suggested a relatively temperate and humid climate in the study area during the early Albian, with an increase of xerophytic vegetation in palynological flora indicating obvious later aridification [31], which is also supported by the discovery of Cheirolepidiaceae macro-fossils from the uppermost Zhonggou Formation [27].

The slender, flexible and upright stems of *Gansufructus* are not lignified, and the longitudinal grooves or ribs on the stem surface probably represent vascular bundles of a herbaceous plant [40,44]. The numerous narrow-lanceolate and alternate leaves attached to the axes and panicle-like infructescence with numerous fruits terminally at the leafy axes would have required stable support and a sufficient vascular system. *Gansufructus saligna* was probably a herbaceous or scarcely woody plant growing in a terrestrial environment. The association with riparian *Equisetum* [28] and fishes suggest a locally wet and lowland environment. Therefore, *Gansufructus* is supposed to be a terrestrial herb colonizing lowland areas, probably growing in the mud or floodplains along lakeshores in a humid environment. The current fossil specimens, together with other taxa recorded from the Jehol Biota and other regions [5,17,35–40,44], indicate the presence of diverse early eudicots of low stature colonizing areas during the middle-late Early Cretaceous.

## MATERIALS AND METHODS

All the fossil specimens were collected from the uppermost Zhonggou Formation of Hanxia Section (39°50^′^ N, 97°15^′^ E), about ∼40 km west of Laojunmiao County, Jiuquan City, Gansu Province (Fig. 1). The fossils were photographed using a SONY Alpha 7 II EOS digital camera coupled with a SONY 50 mm FE macro lens and a stereo microscope (Zeiss, Oberkochen, Germany). The cuticle remains that were removed from the fossil specimens were firstly treated with 10% HCl (hydrogen chloride), and then 50% HF (hydrogen fluoride) subsequently. Some of them were treated with saturated NaClO (sodium hypochlorite), stained in a safranine solution, mounted on slides, embedded in a glycerine jelly, sealed with Canada balsam and then observed under an Axio Scope A1 light microscope and a Stemi508 fluorescence microscope (Zeiss, Oberkochen, Germany) at the Key Laboratory of Petroleum Resources, Gansu Province, Northwest Institute of Eco-Environment and Resources, Chinese Academy of Sciences, Lanzhou, China. Other preparations were mounted on stubs, coated with gold and examined under a JSM-6510 scanning electron microscope (JEOL, Japan) at Lanzhou University, China. All the specimens (specimen no: JQ-2017-01(A, B), JQ-2018-01(A, B), JQ-2018-02(A, B), JQ-2018-03(A, B), JQ-2019-01(A, B), JQ-2020-01 and JQ-2020-02) as well as cuticular slides, were deposited in the Paleontological Laboratory of the School of Earth Sciences, Lanzhou University, China.
